# Estimated Effectiveness of Prior SARS-CoV-2 BA.1 or BA.2 Infection and Booster Vaccination Against Omicron BA.5 Subvariant Infection

**DOI:** 10.1001/jamanetworkopen.2023.2578

**Published:** 2023-03-10

**Authors:** Eun Jung Jang, Young June Choe, Ryu Kyung Kim, Sangwon Lee, Seon Kyeong Park, Young-Joon Park

**Affiliations:** 1 Korea Disease Control and Prevention Agency, Cheongju, South Korea; 2 Korea University Anam Hospital, Seoul, South Korea

## Abstract

This case-control study estimates the effectiveness of prior SARS-CoV-2 BA.1 or BA.2 infection and booster vaccination against Omicron BA.5 subvariant infection.

## Introduction

The booster vaccination against SARS-CoV-2 is effective in preventing COVID-19 and critical infection.^1^ Yet very few studies have investigated the protective effectiveness of prior infection by different Omicron subvariants followed by booster vaccination. In this study, we aimed to estimate protective effectiveness of prior SARS-CoV-2 BA.1 or BA.2 infection and booster vaccination during Omicron BA.5 predominance in South Korea.

## Methods

This nationwide population-based study used a risk-set sampled nested case-control study design. Data were extracted from the Korea COVID-19 Vaccine Effectiveness (K-COVE) data set, described in eMethods in Supplement 1. From the K-COVE cohort, we selected controls who were at risk with the same sex, age, and place of residence (fine matching) (eFigure in Supplement 1).

The study protocol was reviewed and approved by the institutional review board of the Korea Disease Control and Prevention Agency. This study was conducted as a legally mandated public health investigation under the authority of the Korean Infectious Diseases Control and Prevention Act that retrospectively reviewed an anonymized database; therefore, written informed consent was not required. This report follows the Strengthening the Reporting of Observational Studies in Epidemiology (STROBE) reporting guideline for case-control studies.

Cases were individuals who had tested positive for SARS-CoV-2 during the period of BA.5 predominance between August 1 and August 31, 2022. We compared proportions of cases and controls who did not have previous SARS-CoV-2 infection ever and those who had previous BA.1 or BA.2 infections. A logistic regression model was used, and odds ratios (ORs) with 95% CIs from an adjusted model with covariates (health status, vaccination status, infection history) were calculated to estimate vaccine effectiveness (VE) as (1 − OR) × 100.^2^ VE against all BA.5 infections and critical infections by no prior infection, prior BA.1 infection, and prior BA.2 were compared. Statistical analyses were conducted using R version 4.22 (R Project for Statistical Computing).

## Results

A total of 3 415 980 cases and 3 415 980 controls (53.9% were female in both groups; mean [SD] age was 40.2 [21.9] years) were selected, and the Table shows general characteristics of cases and controls. Figure, A shows estimated VE against all BA.5 infections by history of infection and number of vaccinations. In persons without history of SARS-CoV-2 infection, 4-dose estimated VE was 16.1% (95% CI, 15.5% to 16.6%), while in persons with prior BA.1 and BA.2 infections, the estimated VEs were 89.5% (95% CI, 89.2% to 89.8%) and 94.3% (95% CI, 94.1% to 94.4%), respectively. Figure, shows the VE against critical BA.5 infection. Two-dose VEs against critical BA.5 infection was low in persons with no prior infection (41.5%; 95% CI, 32.1% to 49.6%), prior BA.1 infection (53.1%; 95% CI, 15.0% to 74.1%), and prior BA.2 infection (50.0%; 95% CI, −0.29% to 75.0%). 4-dose VEs against critical BA.5 infection was higher at 90.9% (95% CI, 89.8% to 91.9%) in persons without COVID-19 history, and to 93.9% (95% CI, 90.5% to 96.1%) and 92.9% (95% CI, 89.2% to 95.3%) in persons with prior BA.1 and BA.2 infections, respectively.

**Table.** General Characteristics of Cases and Controls

Characteristics	No. (%)	
	Case (n = 3 415 980)	Control (n = 3 415 980)
Sex		
Male	1 573 287 (46.1)	1 573 287 (46.1)
Female	1 842 693 (53.9)	1 842 693 (53.9)
Age, y		
0-4	111 910 (3.3)	111 910 (3.3)
5-11	240 536 (7.0)	240 536 (7.0)
12-17	265 356 (7.8)	265 356 (7.8)
18-39	1 068 360 (31.3)	1 068 360 (31.3)
40-59	986 348 (28.9)	986 348 (28.9)
60-74	535 361 (15.7)	535 361 (15.7)
≥75	208 109 (6.1)	208 109 (6.1)
Place of residence		
Metropolitan	1 597 150 (46.8)	1 597 150 (46.8)
Nonmetropolitan	1 818 830 (53.2)	1 818 830 (53.2)
Health status		
Immunocompromised persons	104 069 (3.0)	93 762 (2.7)
Living in long-term care facility	41 086 (1.2)	31 759 (0.9)
Others	3 270 825 (95.8)	3 290 459 (96.3)
Prior infection and vaccination status^a^		
No prior infection		
2 doses	628 642 (18.4)	414 907 (12.1)
3 doses	1 720 473 (50.4)	1 051 844 (30.8)
4 doses	378 080 (11.1)	315 391 (9.2)
Prior infection (BA.1 period)		
2 doses	24 697 (0.7)	193 849 (5.7)
3 doses	47 924 (1.4)	258 859 (7.6)
4 doses	7397 (0.2)	43 472 (1.3)
Prior infection (BA.2 period)		
2 doses	7045 (0.2)	141 798 (4.2)
3 doses	19 977 (0.6)	270 818 (7.9)
4 doses	4528 (0.1)	49 107 (1.4)

^a^ After exclusion of unvaccinated and 1-dose vaccinated persons.

**Figure.**
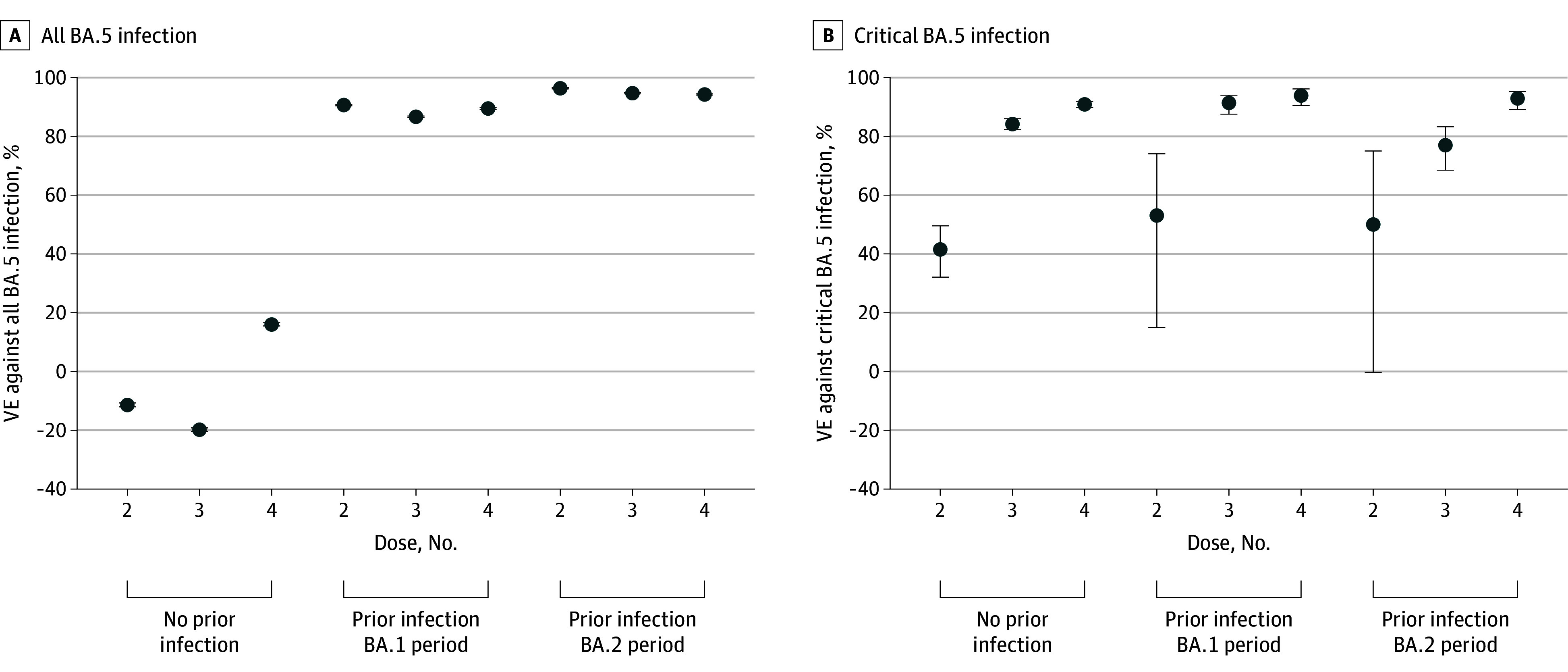
Vaccine Effectiveness (VE) Against All BA.5 Infection and Critical BA.5 Infection Dots indicate point estimate of vaccine effectiveness, and whiskers indicate 95% CI.

## Discussion

Prior SARS-CoV-2 infection during the BA.1 and BA.2 periods was associated with greater protection from SARS-CoV-2 infection during BA.5 period, which is in line with a previous study.^3^ Moreover, protection against critical BA.5 infection was also associated with the number of booster doses, compared with the 2-dose vaccination.^3,4^ The 4-dose booster, irrespective of history of SARS-CoV-2 infection, was associated with higher protection against critical BA.5 infection, as shown in previous studies.^5,6^

There are limitations to this case-control study. Not all positive cases during the observed period may have been identified, and protection from prior infection might be overestimated if exposed people were more likely than unexposed people to get tested. Importantly, the observation that the protection against critical BA.5 infection depends more on 4-dose booster, rather than previous BA.1 or BA.2 infection highlights the importance of booster vaccination.

## Notes

Supplement 1. **eAppendix.** Supplementary Methods
eReferences
**eFigure. **Selection Criteria

Supplement 2.
Data Sharing Statement
