# Supplementary material for: Estimated Effectiveness of Prior SARS-CoV-2 BA.1 or BA.2 Infection and Booster Vaccination Against Omicron BA.5 Subvariant Infection
Source: JAMA Netw Open. 2023 Mar 10;6(3):e232578. doi: 10.1001/jamanetworkopen.2023.2578 (PMC12068789; doi:10.1001/jamanetworkopen.2023.2578)
Supplement: Supplement 1. — eAppendix. Supplementary Methods eReferences eFigure. Selection Criteria [file jamanetwopen-e232578-s001.pdf]

Supplemental Online Content

Jang EJ, Choe YJ, Kim RK, Lee S, Park SK, Park Y-J. Estimated effectiveness of prior SARS-CoV-2 BA.1 or BA.2 infection and booster vaccination against Omicron BA.5 subvariant infection. *JAMA Netw Open*. 2023;6(3):e232578. doi:10.1001/jamanetworkopen.2023.2578

eAppendix. Supplementary Methods

eReferences

eFigure. Selection Criteria

This supplemental material has been provided by the authors to give readers additional information about their work.

© 2023 Jang EJ et al. *JAMA Network Open.*

eAppendix. Supplementary Methods

The study population included 54,040,290 persons registered to the COVID-19 immunization registry by August 2022, and who had residency in Korea since February, 2020.

During the COVID-19 pandemic, all Korean residents had free of access to polymerase chain reaction (PCR), which are registered centrally. Since January 29, 2022, PCR testing was prioritized to those aged 60 years and above, those with COVID-19 suggestive symptoms, those who had epidemiologically-linked to COVID-19 cases, and screening for high-risk group; while those who do not belong PCR priority group, they were freely accessed to rapid antigen test (RAT). The number of PCR and RATs ranged 100,000 to 1,000,000 per day between March 1 and August 31, 2022.[1]

South Korea has had COVID-19 booster vaccination program since April 2022; 3-dose was recommended to all persons aged 12 years and older, while 4-dose were recommended to all persons aged 50 years and older. The booster coverage rate was 70% (33,634,493/47,879,487) for 3-dose and 34% (7,511,472/21,962,768) for 4-dose by October 23, 2022[1]. As of October 20, 2022, type of vaccines was; 67% BNT162b2, 33% mRNA-1273 for 3-dose booster; 87% BNT162b2, 23% mRNA-1273 for 4-dose booster vaccination.[1]

The 3-30% of randomly selected nasopharyngeal specimen collected at the government centers were tested for whole genome sequence for genomic surveillance.[2] Since first identification of Omicron BA.5 in June 2022, it quickly replaced BA.1 and BA.2 and became the predominant subvariant accounting >90% by August 2022. BA.1 predominated in Korea between January 16 and March 19, 2022; while BA.2 predominated between March 20 and July 23.[1,3]

Data were extracted from the Korea COVID-19 Vaccine Effectiveness (K-COVE)

© 2023 Jang EJ et al. *JAMA Network Open.*

dataset maintained at the Korea Disease Control and Prevention Agency (KDCA; Cheongju, Korea), described in detail elsewhere. Briefly, individual-level information is linked daily between national registers, including the national infectious disease surveillance which includes details of all COVID-19 positive persons and vaccine registry. From this dataset , we obtained data on demographic details, date of prior infection, date and doses of vaccination, place of residence, immunocompromised status, long-term care facility center residence, and presence of critical infection defined as hospitalization for COVID-19 with high-flow oxygen therapy, mechanical ventilation, extracorporeal membrane oxygenation, continuous kidney replacement therapy, and death within 28 days of laboratory confirmation of SARS-CoV-2 positivity.

© 2023 Jang EJ et al. *JAMA Network Open.*

eReferences

1. Korea Disease Control and Prevention Agency. Coronavirus 2019 Dashboard. Accessed at: https://ncov.kdca.go.kr/en/

2. Park AK, Kim IH, Kim J, Kim JM, Kim HM, Lee CY, Han MG, Rhie GE, Kwon D, Nam JG, Park YJ, Gwack J, Lee NJ, Woo S, No JS, Lee J, Ha J, Rhee J, Yoo CK, Kim EJ. Genomic Surveillance of SARS-CoV-2: Distribution of Clades in the Republic of Korea in 2020. Osong Public Health Res Perspect. 2021 Feb;12(1):37-43. doi: 10.24171/j.phrp.2021.12.1.06. PMID: 33659153; PMCID: PMC7899228.

3. Lee DW, Kim JM, Park AK, Kim DW, Kim JY, Lim N, Lee H, Kim IH, Kim JA, Lee CY, Kwon JH, Kim EJ. Genomic epidemiology of SARS- CoV-2 Omicron variants in the Republic of Korea. Sci Rep. 2022 Dec 27;12(1):22414. doi: 10.1038/s41598-022-26803-w. PMID: 36575217; PMCID: PMC9793390.

© 2023 Jang EJ et al. *JAMA Network Open.*

eFigure. Selection Criteria

Korea COVID-19 Vaccine Effectiveness (K-COVE) dataset

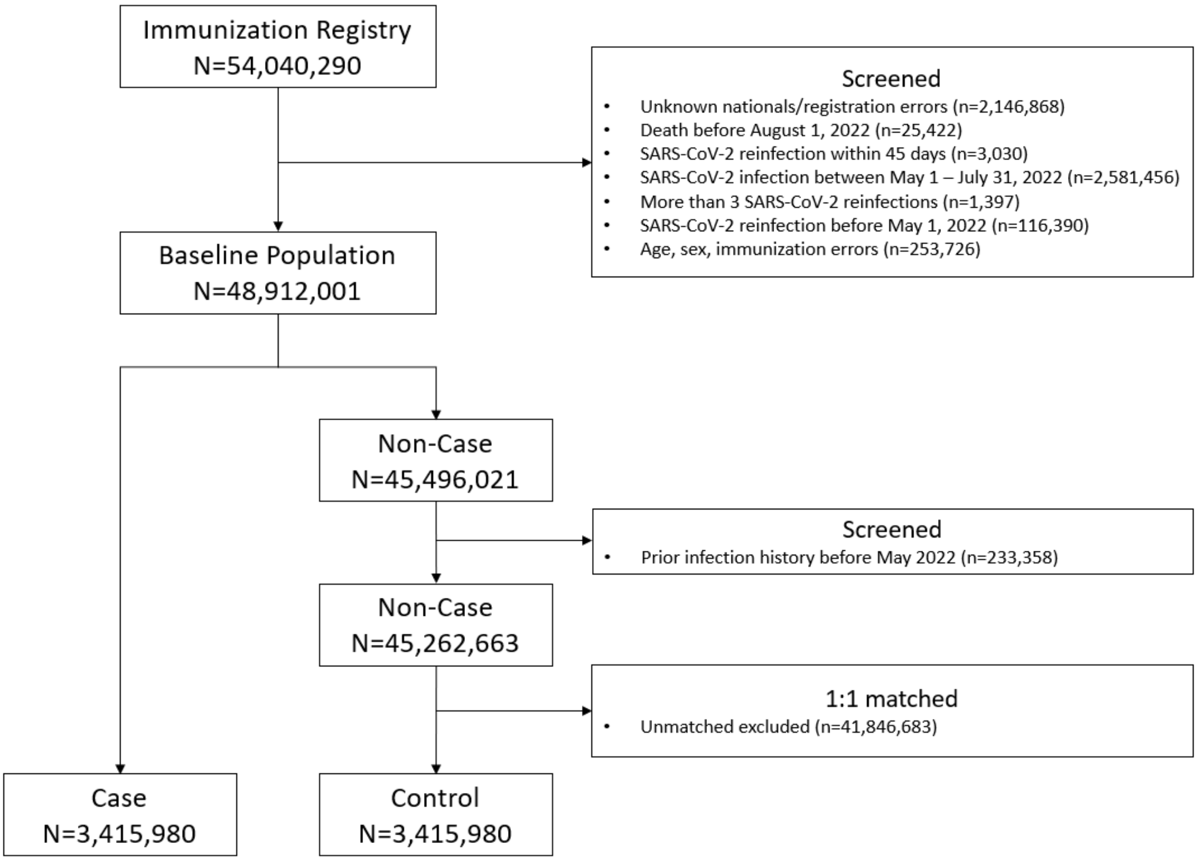

© 2023 Jang EJ et al. *JAMA Network Open.*
