# Supplementary material for: Estimated Effectiveness of Prior SARS-CoV-2 BA.1 or BA.2 Infection and Booster Vaccination Against Omicron BA.5 Subvariant Infection
Source: JAMA Netw Open. 2023 Mar 10;6(3):e232578. doi: 10.1001/jamanetworkopen.2023.2578 (PMC12068789; doi:10.1001/jamanetworkopen.2023.2578)
Supplement: Supplement 2. — Data Sharing Statement [file jamanetwopen-e232578-s002.pdf]

Data Sharing Statement

Jang. Estimated Effectiveness of Prior SARS-CoV-2 BA.1 or BA.2 Infection and Booster Vaccination Against Omicron BA.5 Subvariant Infection. *JAMA Netw Open*. Published March 10, 2023. doi:10.1001/jamanetworkopen.2023.2578

Data
Data available: Yes
Data types: Deidentified participant data
How to access data: pahmun@korea.kr
When available: With publication

Supporting Documents
Document types: Statistical/analytic code
How to access documents: pahmun@korea.kr
When available: With publication

Additional Information
Who can access the data: researchers whose proposed use of the data has been approved
Types of analyses: N/A
Mechanisms of data availability: N/A
